# Time to Leave no Stone Unturned?: Long‐Term Clinical Outcome of d‐SOVP‐Guided Lithotripsy on Quality of Life in Chronic Pancreatitis

**DOI:** 10.1002/ueg2.70106

**Published:** 2025-09-08

**Authors:** Charlotte L. van Veldhuisen, Robert C. Verdonk

**Affiliations:** ^1^ Amsterdam Gastroenterology Endocrinology Metabolism Amsterdam UMC University of Amsterdam Amsterdam the Netherlands; ^2^ Department of Research and Development St Antonius Hospital Nieuwegein the Netherlands; ^3^ Department of Gastroenterology and Hepatology St. Antonius Hospital Nieuwegein the Netherlands

Chronic pancreatitis (CP) is a progressive inflammatory disease of the pancreas, often accompanied by severe abdominal pain and impaired quality of life [1]. Management of CP remains challenging, particularly in relation to pain, due to a complex interplay of several factors. The pathophysiology is multifactorial, involving mechanisms such as inflammation, ductal obstruction, and the formation of pseudocysts, often leading to irreversible destruction of pancreatic parenchyma and loss of function. Over time, fibrosis of the pancreatic parenchyma leads to ductal stenosis, increasing intraductal pressure, which is thought to contribute to visceral pain. Additionally, factor such as psychological distress or depression, feeling of guild and impact on social functioning appear to influence both pain and quality of life.

Typically, management of pain starts with conservative measures (i.e., analgesics) and when these therapies fail, more invasive strategies may be considered. Endoscopic treatment often includes an endoscopic retrograde cholangiopancreatography (ERCP) with sphincterotomy, stent placement, ESWL and stone removal used to alleviate ductal obstruction or pancreatic ductal hypertension [2]. Surgical strategies can be divided into surgical drainage procedures, duodenum‐preserving pancreatic head resections (DPPHR) and formal pancreatic resections [3]. Until recently, a step‐up approach was used, wherein endoscopy‐first was the preferred route and surgery was reserved for more severe and therapy resistant cases. Nevertheless, in recent years, there has been a noticeable trend towards increased use of (early) surgery as first‐line treatment for intractable pain. This shift has been a result of increased evidence by randomized trials of favorable outcomes in surgically treated patients in providing long‐term pain relief [4, 5, 6].

However, one important consideration in evaluating and comparing the efficacy of endoscopic interventions to surgery is that, potentially, earlier studies may have involved sub‐optimal endoscopic techniques. Critics have pointed out that some advanced endoscopic techniques were not included or available in these trials. Recent advancements in pancreatoscopy‐guided lithotripsy are gaining increasing attention within the field [7, 8]. However, many studies only describe technical success or short term outcome.

In this issue of the Journal, Conrad et al. describe the long term follow up after digital‐single‐operator‐video‐pancreatoscopy (d‐SOVP) guided lithotripsy for pancreatic duct stones in 58 patients with chronic pancreatitis [9]. D‐SOVP was evaluated, and proved to be safe and effective in pain control even during long‐term follow‐up. In this, 52 patients (89.7%) showed initial clinical success at 3 months, and persistent clinical success was seen in 41 patients (70.7%) at 24 months‐follow‐up. Yet, no significant influence on quality of life was observed. Also, complete stone removal appeared to be the crucial point for long‐term clinical success.

Particularly in patients with obstructive pancreatic duct stones, and unlike traditional approaches (e.g., ERCP, ESWL), d‐SOVP guided lithotripsy allows for direct intraductal visualization and targeted fragmentation of stones, leading to improved stone clearance. In addition, literature suggest a reduced need for multiple sessions, and clearly, it can be seen as a minimally invasive alternative to surgical intervention [10].

There are however several points that need to be taken into account. First, only a limited number of patients are actual candidates for s‐SOVP when considering anatomy, inflammatory changes and location of stones and strictures. In a recent study form a tertiary center, only 34 of 148 patients referred for endoscopic treatment of chronic pancreatitis were candidates for EHL [8]. Second, in the present work only patients with completely successful initial treatment were include for long term evaluation. Third, in patients with pain, regression to the mean may influence outcome after intervention. This was also highlighted by a recent sham controlled randomized clinical trial [11]. Fourth, it is noticeable that quality of life did not improve despite improvement in pain scores. Finally, one should keep in mind the procedures in this study were done by expert endoscopists.

In summary, while significant progress has been made, challenges persist in the management of CP. The clinical success achieved by dSOVP is comparable or even superior to other, more established, endoscopic and surgical procedures. In light of these findings, one may argue that there is an increasing need for a comprehensive comparative study including “state –of‐the‐art” endoscopy and surgery. Nevertheless, the impact of these interventions on quality of life remains poor thus far. This points out the need for a more individualized, integrated approach, one that considers not only the clinical manifestations (e.g., pain) but also other factors (e.g., psychological and social aspects). Further research is needed to optimize and refine patient selection to achieve improved long‐term outcomes in not online pain, but also quality of life. In this, a patient‐focused approach should be centered, together with a tailored treatment strategy.

## Conflicts of Interest

The authors declare no conflicts of interest.

## Data Availability

Data sharing not applicable to this article as no datasets were generated or analyzed during the current study.
